# New-Onset Intermittent Deceleration-Dependent Left Bundle Branch Block Following Induction of General Anesthesia in a Healthy Patient: A Case Report

**DOI:** 10.7759/cureus.55211

**Published:** 2024-02-29

**Authors:** Sengottaian Sivakumar, Mark J Young, Lazar Popilevsky

**Affiliations:** 1 Anesthesiology, Metropolitan Hospitals, New York, USA; 2 Anesthesiology, Metropolitan Hospital Center, New York, USA

**Keywords:** cardiac electrophysiology, perioperative management, rate-dependent conduction abnormalities, anesthetic agents, intraoperative monitoring, cardiac conduction system, left bundle branch block (lbbb), deceleration-dependent aberrancy (dda)

## Abstract

This case report aims to highlight an atypical presentation of deceleration-dependent aberrancy (DDA) following the induction of general anesthesia in a patient with no known cardiac history. It emphasizes the critical role of intraoperative monitoring and the potential effects of anesthetic agents on the cardiac conduction system.

A 46-year-old Hispanic male with no significant past medical or surgical history presented for surgical repair of a comminuted radial fracture. Following anesthesia induction with propofol, midazolam, and fentanyl, he developed a transient left bundle branch block (LBBB) exhibiting deceleration-dependent characteristics. Despite stable hemodynamics, the LBBB pattern appeared at heart rates below 60 beats per minute and resolved with heart rates above 90 beats per minute. This was managed intraoperatively with glycopyrrolate. Postoperative evaluations, including a 12-lead ECG, echocardiogram, and nuclear stress test, indicated normal biventricular function with a small to moderate reversible perfusion defect. The patient did not report cardiac symptoms postoperatively and did not prefer to undergo a coronary angiogram.

This report underscores the importance of recognizing rate-dependent LBBB as a potential intraoperative complication, even in patients without pre-existing cardiac conditions. The transient nature of DDA, influenced by anesthetic agents and managed through careful monitoring and pharmacological intervention, highlights the necessity for vigilance in perioperative settings. This case contributes to a growing body of evidence suggesting that anesthetic management may require tailored approaches for patients experiencing or at risk for conduction abnormalities.

This case illustrates the complexities of cardiac conduction disturbances such as DDA in the context of general anesthesia, serving as a reminder of the importance of thorough monitoring and the judicious use of rate-modifying drugs. It fosters a deeper understanding of the interaction between anesthesia and cardiac electrophysiology. Further research is needed to explore the mechanisms and management strategies for anesthetic-related cardiac conduction abnormalities.

## Introduction

The left bundle branch block (LBBB) signifies an abnormality in the heart's electrical conduction system, wherein the activation of the left ventricle is delayed [1]. This condition is typically associated with heart disease, including coronary artery disease, cardiomyopathy, and hypertension [2]. Although LBBB is typically associated with an increase in heart rate due to acceleration-dependent aberrancy (ADA), it is uncommonly observed in cases where the heart rate is reduced, a phenomenon referred to as deceleration-dependent aberrancy (DDA) [3]. This case report focuses on an atypical presentation of DDA following the induction of general anesthesia with standard induction agents in a patient with no prior cardiac history. The objective of this report is to underscore the importance of thorough intraoperative monitoring and highlight the potential impact of anesthetic agents on the cardiac conduction system. By scrutinizing such rare clinical scenarios, we can enrich our knowledge and understanding, thereby enhancing patient care during perioperative management. Written Health Insurance Portability and Accountability Act authorization has been obtained from the patient for the publication of this case report. This article also follows the relevant guidelines of Enhancing the Quality and Transparency of Health Research and the 2013 Case Reports Checklist.

## Case presentation

**Figure 1:**
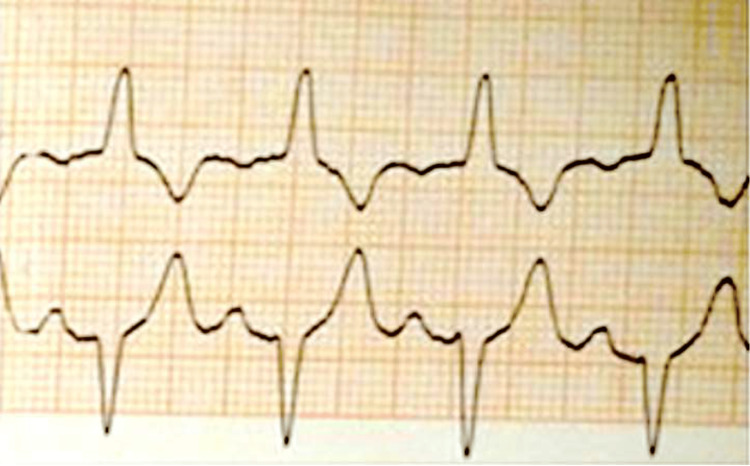
Real-time intraoperative ECG monitoring: printed ECG patterns revealing left bundle branch block.

A 46-year-old Hispanic male with no significant medical history and a BMI of 26.76 kg/m2 presented to the emergency room with severe right wrist pain after a bicycle fall. An X-ray revealed a comminuted fracture of the distal radial metaphysis extending to the radiocarpal joint. He underwent open reduction and internal fixation under general anesthesia with a laryngeal mask airway (LMA) and postoperative supraclavicular block. His pre-induction vitals were blood pressure (BP) of 148/89 mmHg, heart rate of 89 bpm, and oxygen saturation of 100% on room air. Preoperative labs were normal, and he had good effort tolerance (>7 metabolic equivalents (METs)). Standard American Society of Anesthesiologists (ASA) monitors, including a three-lead ECG, were applied, and he received 100% oxygen pre-induction. Anesthesia was induced with propofol 2 mg/kg, midazolam 2 mg, and fentanyl 100 mcg. Soon after induction, lead II ECG showed a widened QRS complex with slurring and notching, concerning for complete LBBB. No prior ECG was available for comparison. His vitals remained stable (BP: 104/65 mmHg; heart rate: 55 bpm). A size 4.0 classic LMA was inserted, and sevoflurane was used for maintenance. ECG patterns were captured from the monitor (Figure 1).

An intraoperative cardiology consult was obtained. The cardiologist recommended that we proceed with the procedure as his vitals were stable. Administration of 200 mcg of glycopyrrolate temporarily raised his heart rate from 55 beats per minute to 92 beats per minute. His LBBB pattern disappeared when his heart rate increased above 90 beats per minute and it reappeared when his heart rate decreased below 60 beats per minute. The phenomenon was observed again and was treated with two more administrations of glycopyrrolate during the surgery. Throughout the entire procedure, his other vital signs, including oxygen saturation and blood pressure, remained normal.

**Figure 2:**
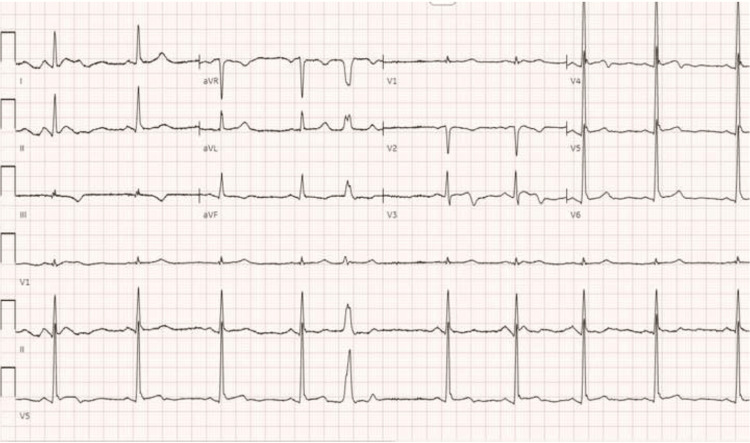
Electrocardiographic findings in the post-anesthesia care unit (PACU): fusion complexes on the 12-lead ECG.

After completion of the surgery, the patient emerged well from anesthesia. His surgical site pain was treated with incremental doses of hydromorphone. A 12-lead ECG in the post-anesthesia care unit (PACU) showed fusion complexes (Figure 2).

**Figure 3:**
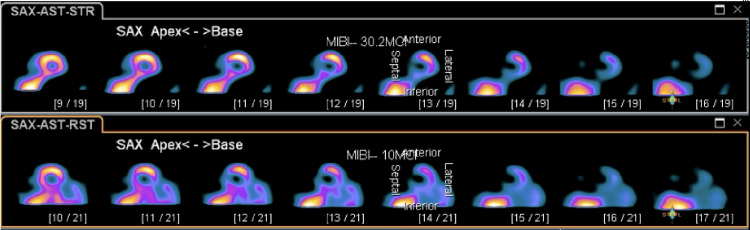
SPECT image 1 showing a small to moderate reversible perfusion defect present in the mid and basal aspect of the inferoseptal wall of the left ventricle. SPECT: single photon emission computed tomography.

**Figure 4:**
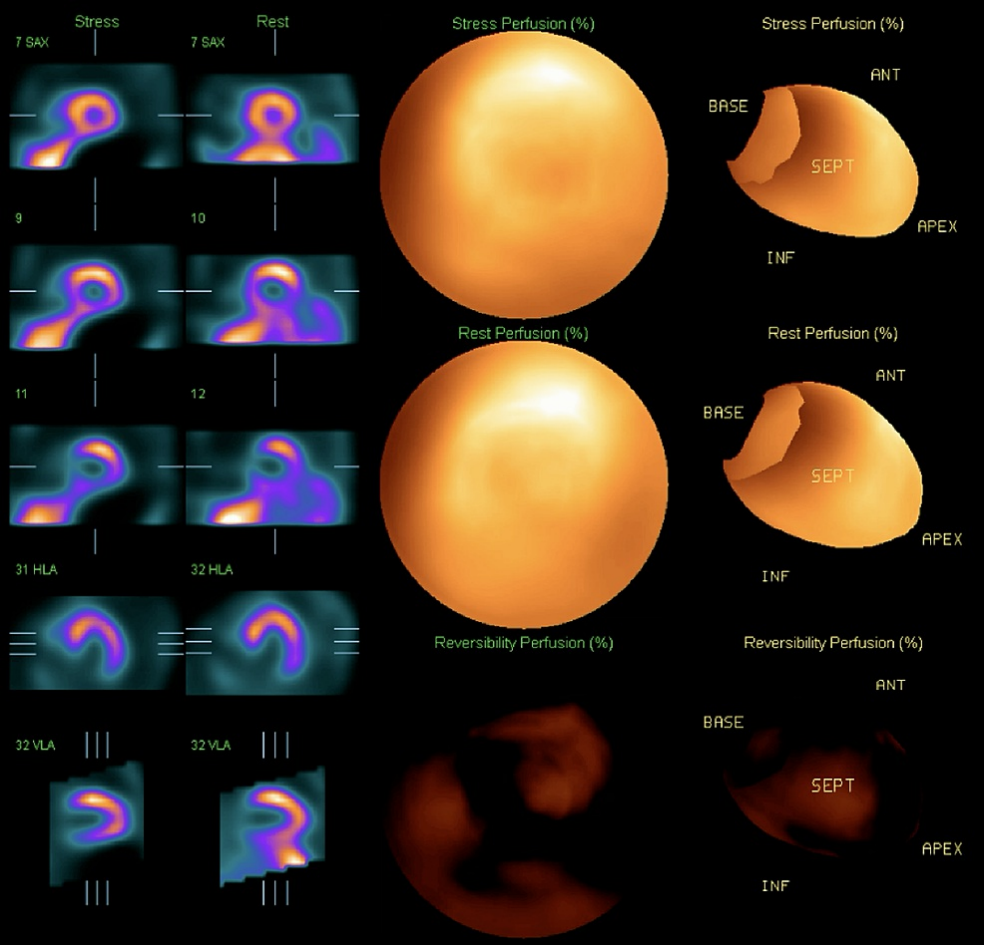
SPECT image 2 showing a small to moderate reversible perfusion defect present in the mid and basal aspect of the inferoseptal wall of the left ventricle. SPECT: single photon emission computed tomography.

**Figure 5:**
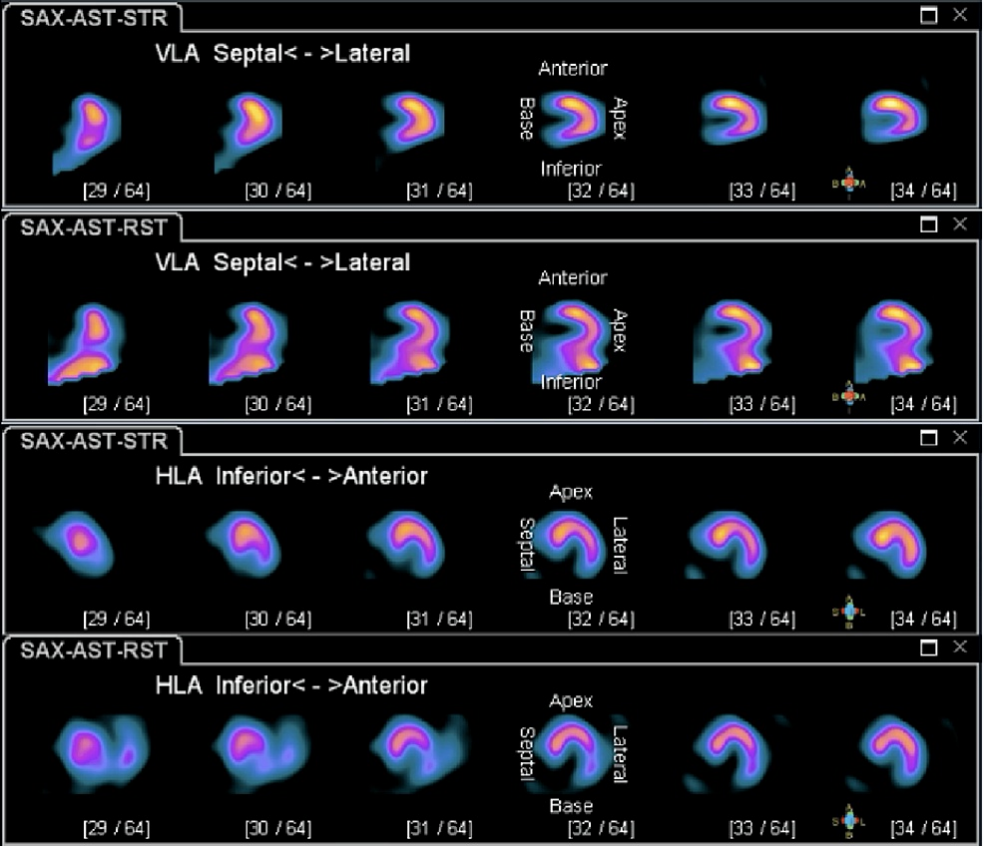
SPECT image 3 showing a small to moderate reversible perfusion defect present in the mid and basal aspect of the inferoseptal wall of the left ventricle. SPECT: single photon emission computed tomography.

A postoperative echocardiogram, also completed in the PACU, showed normal biventricular function. The patient was admitted to the medicine floor for overnight telemetry monitoring. Telemetry captured several LBBB patterns during the nighttime when the patient was sleeping and his heart rate went down to 50-60 beats per minute. The patient never complained of any cardiac symptoms such as chest pain or palpitations. And the vital signs were stable during those episodes. So no treatments were given. An interval nuclear stress test showed a small to moderate reversible perfusion defect present in the mid and basal aspect of the inferoseptal wall of the left ventricle (Figures 3-5).

## Discussion

Although intermittent LBBB may be present in otherwise healthy young adults, it is typically regarded as a benign condition, it frequently manifests in elderly patients with pre-existing conditions, such as hypertension, aortic valvular abnormalities, left ventricular hypertrophy, or coronary artery disease [2]. Additionally, ST segment and T wave abnormalities may be observed. These conditions may be indicative of an occult myocardial or coronary artery disease [2]. Various extrinsic factors, such as hyperkalemia or proarrhythmic drugs, including antiarrhythmics, tricyclic antidepressants, and phenothiazines, can lead to delayed intraventricular conduction as well. Furthermore, the onset of left or right bundle branch block (BBB) can be transient and "rate-related," occurring at either fast or slow heart rates, when the bundle refractory period exceeds the R-R interval [4]. Tachycardia-related LBBB, also known as ADA or "phase 3 block," originates from a defect in phase 3 (repolarization) at higher heart rates. This defect delays or completely blocks impulses coinciding with the refractory period of the action potential. Conversely, bradycardia-related LBBB, termed DDA or "phase 4 block," is linked to the spontaneous diastolic depolarization of diseased Purkinje cells. Unlike their healthy counterparts, these diseased cells exhibit phase 4 depolarization [5].

When an impulse from either the supraventricular or ventricular system reaches these cells during phase 4, sodium channels are inactive, preventing depolarization and leading to asystole. Conduction will only resume with an appropriately timed escape beat or premature beat, which resets the transmembrane potential to its maximum resting value. This is often induced by spontaneous sinus rate slowing or pauses triggered by atrial or ventricular premature beats [4]. Additional complexity arises from contributing mechanisms like the linking phenomenon and refractory period mismatch [6]. The linking phenomenon [7] specifically describes the mechanism that perpetuates functional anterograde BBB through repetitive transseptal retrograde concealed penetration by impulses propagating along the contralateral bundle. Refractory period mismatch occurs when the refractory periods between different parts of the conduction system are misaligned, making the system prone to reentrant circuits. Another mechanism, enhanced phase 4 depolarization, comes into play when the slowed heart rate prolongs phase 4 of the cardiac action potential, making the left bundle branch more susceptible to blockage. Rate-dependent LBBB has been associated with acute decreases in systolic cardiac function [8], and instances where both ADA and DDA mechanisms coexist in a single patient have been documented [9]. This adds another layer of complexity to the diagnosis and management of the condition. Despite chronic BBB's link to postoperative myocardial infarction (MI), its presence on preoperative ECG does not improve risk prediction beyond a history of ischemic heart disease. Chronic hemiblocks also do not alter anesthetic plans, but a new hemiblock requires further evaluation [10]. Recent research confirms BBB's association with postoperative MI but emphasizes the superiority of clinical risk factors for prediction [11]. Thus, clinical assessment outweighs ECG findings. However, an LBBB warrants long-term cardiology care due to elevated cardiovascular mortality risks [12].

Volatile anesthetics have the potential to impact sinoatrial node discharge rates [13] and alter ion channel functions [14], which could be particularly relevant in cases of ADA and DDA. For ADA, the goal is to prevent tachycardia by minimizing sympathetic stimulation, particularly during critical phases like laryngoscopy or surgical incision. Intravenous agents known for their sympathomimetic or vagolytic activities, such as ketamine and anticholinergics, should be used sparingly and in carefully titrated doses. For DDA, the strategy shifts toward avoiding exacerbation of bradycardia. Drugs that significantly reduce heart rate, like alpha-2 agonists, beta-blockers, or high doses of opioids, should be used cautiously. Likewise, it is important to anticipate and manage situations that might trigger vagal stimulation, using agents like atropine in a carefully titrated manner. Maintaining an appropriate depth of anesthesia is crucial to avoid further complications related to bradycardia. This tailored approach ensures that the challenges presented by either ADA or DDA are adequately managed. In cases where a pulmonary artery catheter is necessary for surgery, anesthesia providers should be aware of the risk of transient right bundle impairment, potentially causing a complete heart block. Therefore, immediate availability of a temporary transcutaneous or transvenous pacemaker should be ensured before anesthesia induction. If a point of care ultrasound (POCUS) echocardiogram were done, the parasternal long-axis view would reveal the dynamic posterior motion of the interventricular septum within 0.04 seconds of QRS onset, preceding the anterior motion of the posterior left ventricular wall during ejection, offering additional diagnostic insight into LBBB [15].

## Conclusions

In summary, this case highlights the critical need for careful intraoperative monitoring and cautious use of rate-modifying drugs. It shows that new or acute BBBs warrant investigation, even in asymptomatic patients with no cardiac history. While the case did not necessitate postponing low-risk surgery for further evaluation, it raises questions about the impact of medications on cardiac conduction and the potential for transient ischemic events in those without traditional risk factors. Though limited by its unique circumstances and lack of invasive tests, this case serves as a valuable catalyst for further research into these rare but significant cardiac anomalies.
